# Radiomic-enhanced multimodal ultrasound for early detection of acute kidney injury secondary to renal vein thrombosis: a preclinical diagnostic modeling study

**DOI:** 10.1080/0886022X.2025.2525472

**Published:** 2025-07-01

**Authors:** Ziyi Xu, Xinghua Wang, Nan Qiao, Tao Zhang

**Affiliations:** a Department of Ultrasound, Shanxi Provincial People’s Hospital Affiliated to Shanxi Medical University, Taiyuan, China; b Departments of Ultrasound, Second Hospital of Shanxi Medical University, Taiyuan, China; c Shanxi Province Cancer Hospital/Shanxi Hospital Affiliated to Cancer Hospital, Chinese Academy of Medical Sciences/Cancer Hospital Affiliated to Shanxi Medical University, Taiyuan, China; d Medical Imaging Department of Shanxi Medical University, Taiyuan, China

**Keywords:** Multimodal, ultrasound, radiomics, acute kidney injury, acute renal vein thrombosis

## Abstract

Acute kidney injury (AKI) resulting from acute renal vein thrombosis (ARVT) is uncommon, yet it can progress swiftly, requiring prompt diagnosis and intervention. This study aimed to investigate the various multimodal ultrasound techniques, specifically conventional ultrasound (CUS), microvascular flow imaging (MFI), contrast-enhanced ultrasound (CEUS), and shear wave elastography (SWE), in conjunction with radiomics for early diagnosis and assessment of AKI resulting from ARVT using a rabbit model. Twenty healthy adult New Zealand white rabbits with 40 kidneys were included in this study. The left kidneys were designated as the experimental group (*n* = 20), whereas the right kidneys served as the control group(*n* = 20). Throughout the study, multimodal ultrasound techniques were employed for image acquisition and analysis. The ultrasound images underwent processing, segmentation, feature extraction, and model construction. The dataset was randomly divided in a 7:3 ratio, and the performance of models was assessed through the Receiver Operating Characteristic Curve (ROC) analysis along with key performance metrics. In CUS images, the experimental group showed notable increases in renal volume, cortical thickness, and enhanced cortical echogenicity (*p* < 0.001, *p* = 0.032, *p* < 0.001). In the CDFI, MFI, and CEUS, the experimental group exhibited significant reductions in blood flow perfusion (*p* < 0.001). In SWE, Young’s modulus values for the cortex, medulla, and sinus were significantly elevated in the experimental group (*p* < 0.001). The strongest correlations were found for creatinine, renal volume, peak systolic velocity of the arcuate artery, time from peak to half-value of sinus, and Young’s modulus value for cortex minimum, with Area Under the Curve(AUC) values of 0.600, 0.868, 0.560, 0.503, and 0.982, respectively. The CUS, CDFI, MFI, CEUS, SWE, and CUS+CDFI+MFI+CEUS+SWE radiomics models demonstrated stronger performance, achieving AUC values of 0.899, 0.861, 0.899, 0.833, 0.861, and 0.734, respectively. Multimodal ultrasound combined with radiomics can significantly improve early diagnosis of AKI following ARVT, providing valuable insights for clinical research.

## GRAPHICAL ABSTRACT

**Figure:**
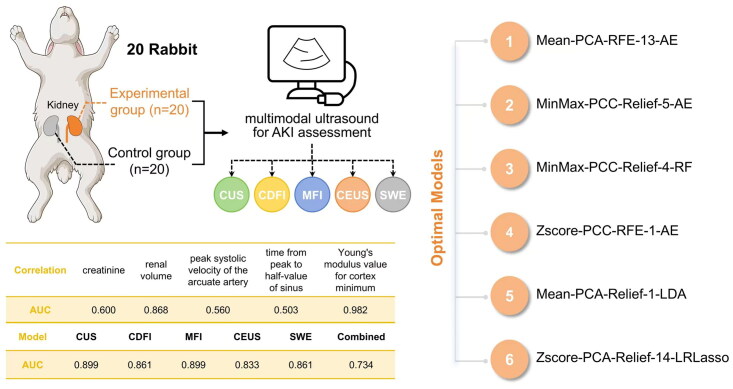

## 1. Introduction

Acute Kidney Injury (AKI) caused by Acute Renal Vein Thrombosis (ARVT) is a pathological condition in which thrombosis of the renal veins, obstructs blood flow, raises renal venous pressure, and impairs glomerular and tubular perfusion, ultimately resulting in renal dysfunction [1–3]. Common causes include renal diseases (e.g., renal tumors), hematological disorders (e.g., thrombotic conditions), anatomical abnormalities in the renal vein (e.g., venous variations), drug-related factors, and surgical interventions or traumatic injury [4–6]. Although ARVT is relatively rare, it can progress rapidly, necessitating timely diagnosis and treatment. Current diagnostic methods primarily rely on imaging techniques such as renal venography, contrast-enhanced CT, and MRI [7]. Despite being effective, these methods have limitations, including invasiveness, exposure to ionizing radiation, nephrotoxicity of contrast agents, and high cost [8]. Ultrasound offers a promising alternative. Advancements such as microvascular flow imaging (MFI), contrast-enhanced ultrasound (CEUS), and shear wave elastography (SWE) make it a noninvasive accessible tool capable of providing real-time, high-resolution images for early diagnosis and monitoring [9–11]. While ultrasound provides valuable qualitative data, it often lacks the capacity to detect complex patterns and subtle variations in tissue characteristics that are crucial for precise diagnosis.

Radiomics, a field that enables high-throughput extraction of quantitative features from medical images, has emerged as a promising method to enhance ultrasound diagnostics [12]. By analyzing a broad range of image features such as texture, shape, and statistical patterns, radiomics provides deeper insights into tissue heterogeneity, vascular changes, and renal function [13]. Few studies have currently explored the use of multimodal ultrasound in the assessment of *in vivo* AKI resulting from ARVT. To address this gap, the present study utilized a rabbit model to examine renal and venous characteristics to assess *in vivo* AKI resulting from ARVT. This prospective study integrates radiomics with multiple ultrasound modalities, including CUS, CDFI, MFI, CEUS, and SWE. This research aims to build diagnostic models based on distinct imaging features, providing experimental evidence that supports clinical translation and early detection of ARVT-induced AKI.

## 2. Materials and methods

### 2.1. Establishment of animal model

**Figure 1.**
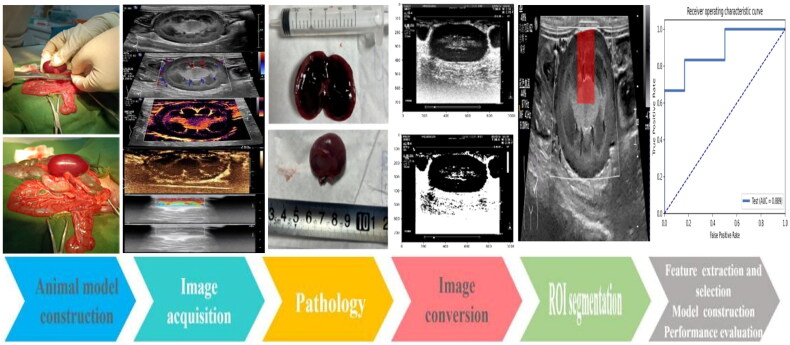
Flowchart of the overall experimental process. We first established an experimental rabbit model of acute renal vein thrombosis was established. Second, multimodal ultrasound images were obtained. Subsequently, the animal specimens were subjected to pathological analysis. Finally, the images were converted, segmented for the region of interest, followed by feature extraction and selection, the establishment of radiomic model, and performance evaluation.

This prospective study was approved by the Institutional Animal Care and Use Committee of our institution (Approval No. 2023-075). A flowchart illustrating the study design is shown in Figure 1.

#### 2.1.1. Animal model selection

A total of 22 healthy adult New Zealand white rabbits (weight range: 1.75–3.56 kg) were initially enrolled. However, one rabbit died due to a stress response and another to an anesthetic overdose, resulting in a final cohort of 20 rabbits. The rabbits were evenly divided by sex (16 males and 4 females), providing a total of 40 kidneys. After careful consideration of the anatomical characteristics, the left kidneys were assigned to the experimental group (*n* = 20), while the right kidneys served as the control group (*n* = 20). Prior to the procedure, the rabbits were acclimated to laboratory conditions for a specified period, under standard lighting and an ambient temperature of approximately 25 °C.

#### 2.1.2. Induction of acute renal vein thrombosis

1. Experimental Group: The rabbits were administered respiratory anesthesia and securely positioned on the operating table. Following aseptic preparation, an 8-cm midline incision was made along the abdomen. The intestines were retracted to the right and covered with warm physiological saline. The left renal artery, vein, and ureter were carefully identified and isolated. A 3-0 suture was tied around the proximal left renal vein, approximately 1 cm from the renal hilum. The abdominal cavity was irrigated with warm saline and the intestines were repositioned. The abdominal layers were closed using standard surgical closure techniques.
2. Control Group: No surgical intervention or modification was performed in the control group.
3. The success of the AKI model was confirmed via histopathological analysis of renal tissue, using the Kidney Histological Injury Score to evaluate injury severity [14].

### 2.2. Multimodal ultrasound scanning protocol

#### 2.2.1. Instrumentation and contrast agents

The Philips EPIQ 7 Color Doppler Ultrasound System (Philips, Amsterdam, Netherlands), equipped with an eL18-4 linear array transducer operating within a frequency range of 4–18 MHz was utilized for CUS, CDFI, MFI, and CEUS. The AixPlorer Color Doppler Ultrasound System (SuperSonic Imagine, Aix-en-Provence, France), fitted with a 15–4 linear array transducer operating at a frequency range of 4–15 MHz was employed for SWE. Sulfur hexafluoride microbubbles (Bracco, Milan, Italy) was used as the contrast agent. All procedures followed the American College of Veterinary Radiology (ACVR) and the European College of Veterinary Diagnostic Imaging (ECVDI) for standardized abdominal ultrasound in veterinary imaging [15].

#### 2.2.2. Measurement methods and observational parameters for CUS, CDFI, and MFI

Two hours post-modeling, the rabbits were anesthetized (Zoletil 50, dosage of 0.1 mL/kg) and positioned supine. The abdominal area was prepared for imaging, and CUS was used to measure renal volume (*V* = 0.49 × length × width × thickness), cortical thickness, and changes in cortical echogenicity. Echogenicity was graded as Grade I (normal cortex), Grade II (increased echogenicity relative to renal sinus but lower than the normal renal pelvis), and Grade III (similar to the normal renal pelvis) [16]. Subsequently, CDFI and MFI were employed to observe the distribution of blood flow within the main trunk and branches of the renal artery and renal vein. Qualitative analysis was performed to determine renal perfusion status [16], categorized as Grade I, denoting normal renal perfusion with blood flow signals extending beneath the renal capsule; Grade II, indicating poor renal perfusion with signals reaching the renal cortex but not extending beneath the renal capsule; and Grade III, indicating severe impairment of renal perfusion with no signals reaching the renal cortex. Pulsed-wave (PW) measurements, including peak systolic velocity (Vmax) and resistance index (RI), were measured in the main trunk and branches of the renal artery and renal vein. Similar measurement methods were applied in the control group.

#### 2.2.3. Measurement methods and observational parameters for CEUS

1. Examination methodology: Prior to model establishment, a catheter was implanted into the left front leg of each rabbit to enable subsequent ultrasound contrast imaging, which was conducted 2 hours after model induction. The mechanical index (MI) was set to <0.1. Before injection, the contrast agent freeze-dried powder was mixed thoroughly with 5 mL of physiological saline and agitated vigorously. It was then administered via the intravenous bolus method at a dose of 0.1 mL/kg, followed by a rapid flush with 1.5 mL of physiological saline. The examination duration lasted for 5 minutes after injection of the contrast agent, and all contrast images were saved for subsequent analysis.
2. Quantitative analysis of images: Upon completion of the contrast imaging, a region of interest (ROI) sampling frame from the upper, middle, and lower parts was selected using time-intensity curve (TIC, version 6.3.3, Philips, Amsterdam, Netherlands) analysis software, and placed specifically within the cortex, medulla, and sinus. Subsequently, TICs were then generated for each sample, ensuring that the ROIs for different samples were consistently positioned at the same depth whenever possible. The analyzed parameters encompassed the following: ① Curve slope of ascending (CSA); ② Time to peak (TTP); ③ Peak intensity (PI); ④ Area under the curve (AUC); ⑤ Mean transit time (MTT); ⑥ Time from peak to half value (T_1_/_2_); ⑦ Rise time (Tr). Finally, a comparative analysis was performed by calculating the average values for the upper, middle, and lower regions of the cortex, medulla, and sinuses.

#### 2.2.4. Measurement methods and observational parameters for SWE

Observations were conducted 2 h after model induction. Elastographic imaging mode was activated and the ultrasound beam was directed toward the renal hilum, perpendicular to the lateral side of the kidney. SWE images of the cortex, medulla, and sinus from the upper, middle, and lower regions were captured. Once the images stabilized, the average, minimum, maximum, and standard deviation values of Young’s modulus were measured. The measurement range (Q-Box diameter) was maintained at a fixed diameter of 2 mm for all assessments. Three measurements were taken, and the average of these measurements was used for statistical analysis. To ensure consistency across measurements, efforts were made to maintain the same position during measurements across different time points and samples.

### 2.3. Laboratory and histopathological examination

On the day of modeling, blood samples were drawn from the left forelimb of the rabbits. Laboratory tests included total protein (TP: 55–72 g/L), albumin (ALB: 25–48 g/L), blood urea nitrogen (BUN: 3.6–8.6 mmol/L), creatinine (CRE: 71–159 µmol/L). Subsequently, under sterile conditions, bilateral kidneys along with their corresponding renal veins were harvested for histopathological examination. The specimens were fixed in 10% formalin solution, dehydrated, paraffin-embedded, sectioned, and stained with hematoxylin and eosin (HE) to prepare histopathological slides. Microscopic observation was carried out using a light microscope (Olympus, Olympus Corporation, Tokyo, Japan). The Kidney Histological Injury Score (KHIS) [14] was used to assess the severity of acute kidney injury. Each category was scored based on histological observations of the kidney tissue, and the total score reflects the severity of the kidney injury. A higher total score implied more severe damage.

**Content of Kidney Histological Injury Score (KHIS)**

**Table:** 

Score Category	Score Criteria	Description
1. Tubular Injury	0	No tubular injury
	1	Mild injury (e.g., slight tubular vacuolization or shedding)
	2	Moderate injury (e.g., moderate tubular necrosis, dilation, or debris)
	3	Severe injury (e.g., extensive tubular necrosis, structural loss)
2. Interstitial Inflammation	0	No inflammation
	1	Mild inflammation (e.g., scattered inflammatory cells in the interstitium)
	2	Moderate inflammation (e.g., dense inflammatory cell infiltration in the interstitium)
	3	Severe inflammation (e.g., widespread inflammatory cell infiltration with fibrosis)
3. Glomerular Injury	0	Normal glomeruli
	1	Mild injury (e.g., slight mesangial expansion or mild sclerosis)
	2	Moderate injury (e.g., partial sclerosis or necrosis of glomeruli)
	3	Severe injury (e.g., widespread sclerosis or necrosis of glomeruli)
4. Vascular Injury	0	Normal vascular structure
	1	Mild injury (e.g., endothelial swelling or mild thickening of the vessel wall)
	2	Moderate injury (e.g., moderate congestion or thrombosis in the vessels)
	3	Severe injury (e.g., widespread thrombosis or vascular necrosis)

**Scoring criteria of Kidney Histological Injury Score (KHIS)**

**Table:** 

Severity of Injury	Total Score	Description
Mild Injury	0–4	Localized injury, usually does not affect renal function, with minor tissue damage.
Moderate Injury	5–7	Larger areas of damage may impact renal function, requiring further observation and management.
Severe Injury	8–12	Extensive damage may significantly affect renal function, requiring timely intervention to reduce damage.

### 2.4. Processing flow of radiomics

#### 2.4.1. Image processing and segmentation

First, using PyCharm software (PyCharm Community Edition 2023.3.5, JetBrains, Prague, Czech Republic), the ultrasound images from CUS, CDFI, MFI, CEUS, and SWE were processed, converted to grayscale, and binarized to standardize the image; then, the processed images were converted to DICOM format using 3D Slicer software (version 5.6.1, Brigham and Women’s Hospital Surgical Planning Laboratory & MIT Artificial Intelligence Laboratory, Boston, MA, USA). Subsequently, segmentation was performed using ITK-SNAP software (version 4.0.1, University of Pennsylvania Penn Image Computing and Science Laboratory, Pennsylvania, USA). Two ultrasound physicians with over 10 years of experience segmented the same image set, achieving strong agreement (Cohen’s kappa = 0.776). Each image was divided into six regions, namely: upper lateral, middle lateral, lower lateral, upper medial, middle medial, and lower medial. A total of 1200 ultrasound images were collected from different modalities, with 600 images for both the experimental and control groups. Each modality contributed an average of 240 images, and each renal model yielded approximately 30 images. Both the original and segmented images were preserved for analysis and documentation.

#### 2.4.2. Feature extraction, merging, preprocessing, and model construction

Feature extraction was conducted using the open-source software FeAture Explorer (FAE, version 0.5.8, Shanghai Key Laboratory of Magnetic Resonance, East China Normal University, Shanghai, China) in Python (version 3.7.6, Python Software Foundation, Beaverton, OR, USA), with an image normalization scale set to 1000. Discretization was performed with a Bin Width of 25.00 and a Bin Count of 16. The image types included original, wavelet transform, square, square root, logarithm, Laplacian of Gaussian, gradient, exponential, and local binary patterns. The extracted features included first-order, shape-based, and second-order parameters that mainly involved the Gray Level Co-Occurrence Matrix (GLCM), Gray Level Run Length Matrix (GLRLM), Gray Level Size Zone Matrix (GLZSM), Neighboring Gray Tone Difference Matrix (NGTDM), and Gray Level Dependence Matrix (GLDM). The dataset extracted from the ultrasound images was collected using five different ultrasound modalities (CUS, CDFI, MFI, CEUS, and SWE), with an average of 240 images per modality, divided into two groups: 120 images for the positive group and 120 images for the negative group. The dataset was randomly split at a 7:3 ratio, with 70% used for the training set (*n* = 168, positive/negative = 84/84) and 30% for the test set (*n* = 72, positive/negative = 36/36), to prevent overfitting. During model construction, data balancing was set to zero. Using MinMax, Z-Score, and Mean normalization procedures, all US radiomic features were normalized and the preprocessing step selected PCC and PCA, with the former set to 0.99 to ensure high consistency among the extracted features, and the latter utilized for dimensionality reduction, with the number of selected features ranging from 1 to 20. Feature selection methods included ANOVA, KW, RFE, and Relief. Based on the final screened radiomic features, machine learning models were constructed by SVM, AE, LDA, Random Forest, Logistic Regression, LR-Lasso, Adaboost, Decision Tree, Gaussian Process, and Naive Bayes. Ten-fold cross-validation was employed to enhance the repeatability and robustness of features to improve model performance.

Model performance was evaluated using receiver operating characteristic (ROC) curve analysis. The area under the ROC curve (AUC) was calculated, and the accuracy (ACC), sensitivity (SEN), specificity (SPE), positive predictive value (PPV), and negative predictive value (NPV) were also calculated at a cutoff value that maximized the value of the Yorden index.

### 2.5. Statistical analyses

All statistical analyses were performed using IBM SPSS Statistics for Windows version 26.0 (IBM Corp., Armonk, NY, USA). *p* values <0.05 were considered statistically significant. Data normality was assessed *via* the Kolmogorov–Smirnov test. Quantitative data with normal distribution are expressed as mean ± standard deviation and assessed using an independent samples t-test, while quantitative data with non-normal distribution are expressed as median ± interquartile intervals and evaluated using the Mann–Whitney *U* test. At the same time, ordinal data were reported as numbers and percentages and analyzed with the Mann–Whitney *U* test for univariate analysis. The clinical model used logistic regression for variable selection and was assessed using ROC curve analysis.

## 3. Results

### 3.1. Rabbit models and clinical characteristics

A total of 20 rabbits were included in this study, with body weights ranging from 1.75 to 3.56 kg (2.5 ± 0.46 kg, mean ± SD). Among the animals, total protein (TP) levels were elevated in four rabbits (20%) and decreased in one (5%). Albumin (ALB) levels were reduced in one rabbit (5%). Blood urea nitrogen (BUN) levels were elevated in 13 rabbits (65%). Creatinine (CRE) levels were increased in three rabbits (15%) and decreased in two rabbits (5%). Detailed clinical parameters are summarized in Table 1.

**Table 1.** Baseline characteristics of participants.

			Laboratory examinations			
Number	Sex	Weight (kg)	TP (g/L)	ALB (g/L)	BUN (mmol/L)	CRE (µmol/L)
1	M	2.48	67.60	42.90	10.50^a^	153.00
2	M	2.60	78.20^a^	39.90	8.73^a^	57.00^b^
3	M	2.58	75.10^a^	40.50	7.81	74.00
4	M	3.00	63.10	40.90	7.35	89.00
5	F	2.30	74.20^a^	41.60	5.27	80.00
6	M	2.25	56.20	30.10	5.71	120.00
7	F	3.00	38.70^b^	19.10^b^	19.50^a^	191.00^a^
8	M	3.20	58.60	32.40	7.14	92.00
9	M	2.50	61.20	38.10	10.00^a^	117.00
10	M	1.75	57.10	38.00	8.90^a^	125.00
11	F	1.90	71.30	42.10	7.02	80.00
12	F	1.75	67.40	39.60	10.20^a^	118.00
13	M	2.25	61.30	29.60	10.80^a^	114.00
14	M	2.75	57.20	38.80	10.00^a^	84.00
15	M	2.35	58.20	41.90	8.78^a^	55.00^b^
16	M	2.43	58.40	37.30	8.07	105.00
17	M	3.56	67.80	39.10	10.80^a^	180.00^a^
18	M	2.78	58.30	26.80	10.20^a^	133.00
19	M	2.95	57.00	31.50	11.60^a^	172.00^a^
20	M	2.30	55.60	33.50	9.06^a^	117.00
$\bar{X}\pm S$		2.53 ± 0.46				112.80 ± 38.68
*M ± QR*			59.90 ± 10.63	38.45 ± 9.07	8.98 ± 2.96	

TP: total protein; ALB: albumin; BUN: blood urea nitrogen; CRE: creatinine.

^a^above the normal range; ^b^below the normal range; M: male; F: female.

### 3.2. Comparison and performance of multimodal ultrasound

CUS revealed significantly increased renal volume, cortical thickness, and enhanced cortical echogenicity in the experimental group compared to controls (*p* < 0.001, *p* = 0.032, *p* < 0.001). The main stem veins were dilated with thrombi confined to the main trunk, presenting as areas of low echogenicity. The narrowing rate of the thrombus area in the main stem veins was 95.6 ± 3.9%, and no significant blood flow signals were observed. PW Doppler demonstrated significantly reduced imaging revealed that, compared to the control group, the peak systolic velocity in the arteries and veins of the main stem, intersegmental, interlobar, and arcuate regions of the experimental group was significantly reduced, with a statistically significant difference between the two groups (all of *p* < 0.001). Spectral analysis showed a high resistance index (RI) in the experimental group, evident through diastolic flow reversal or a high-resistance pattern, whereas the control group consistently showed low-resistance flow. CDFI, MFI, and CEUS each revealed significantly reduced perfusion in the experimental group: all 20 cases on CDFI were classified as grade III (*p* < 0.001); MFI showed 6 cases at grade II, and 14 cases at grade III (*p* < 0.001); CEUS revealed 18 cases at grade II and 2 cases at grade III (*p* < 0.001). In the experimental group, Time-intensity curve (TIC) parameters such as CSA(cortex), Tr (cortex), TTP (medulla), and MTT (sinus), showed no significant differences from the control group (*p* > 0.05), while other parameters differed significantly (*p* < 0.05). SWE showed significantly higher Young’s modulus values for the cortex, medulla, and sinus in the experimental group; all were significantly different from those in the control group (*p* < 0.001). Comprehensive details are provided in Table 2.

**Table 2.** Comparison and performance of multimodal ultrasound.

	Experimental group	Control group	*t*	Z	*p*
**CUS**					
volume (cm^3^)	22.73 ± 6.42	13.80 ± 3.55	5.443		<0.001
cortical thickness (cm)	0.36 ± 0.05	0.32 ± 0.03	2.229		0.032
cortical echogenicity (N)					
I	0	20		−5.941	<0.001
II	14	0			
III	6	0			
width of MRV (cm)	0.44 ± 0.08	0.28 ± 0.09			<0.001
width of MRA (cm)	0.17 ± 0.03	0.16 ± 0.04			0.383
**CDFI**					
Grade (N)					
I	0	20		−6.245	<0.001
II	0	0			
III	20	0			
**PW[Vmax (cm/s)]**					
main stem					
artery	18.28 ± 9.99	58.82 ± 15.66	−9.759		<0.001
vein	0	28.65 ± 11.19			<0.001
intersegment					
artery	7.68 ± 5.66	31.25 ± 16.13		5.415	<0.001
vein	2.23 ± 5.13	15.25 ± 17.72		5.153	<0.001
interlobar					
artery	5.02 ± 7.71	23.46 ± 7.25		5.432	<0.001
vein	0.00 ± 2.41	9.56 ± 6.33		5.357	<0.001
arcuate					
artery	0	11.25 ± 5.58		5.783	<0.001
vein	0	5.99 ± 2.03		5.783	<0.001
**MFI**					
Grade (*N*)					
I	0	20		−5.941	<0.001
II	6	0			
III	14	0			
**CEUS**					
Grade (*N*)					
I	0	20		−6.109	<0.001
II	18	0			
III	2	0			
TIC					
Cortex					
CSA (dB/s)	2.21 ± 26.20	1.39 ± 3.40		−0.582	0.565
TTP (s)	34.07 ± 15.07	25.18 ± 5.85	2.457		0.021
PI (dB)	11.16 ± 10.82	16.52 ± 39.80		2.719	0.006
AUC (dB s)	651.68 ± 434.66	1206.98 ± 2188.11		3.273	0.001
MTT (s)	28.40 ± 19.97	36.45 ± 9.31		2.976	0.002
T₁/₂ (s)	34.20 ± 44.33	55.11 ± 14.55		3.057	0.002
Tr (s)	9.79 ± 10.05	10.12 ± 3.8		1.298	0.201
Medulla					
CSA (dB/s)	2.97 ± 9.56	1.17 ± 1.84		−2.151	0.030
TTP (s)	29.77 ± 19.86	27.04 ± 7.56		−1.325	0.192
PI (dB)	14.86 ± 16.77	18.63 ± 42.30		2.083	0.038
AUC (dB s)	735.38 ± 460.32	1155.24 ± 2278.80		3.273	0.001
MTT (s)	29.27 ± 17.62	36.91 ± 8.83		2.110	0.035
T₁/₂ (s)	30.04 ± 16.25	53.57 ± 12.21	−5.176		<0.001
Tr (s)	4.85 ± 10.92	11.64 ± 3.21		2.570	0.009
Sinus					
CSA (dB/s)	16.75 ± 33.20	1.14 ± 2.07		−2.706	0.006
TTP (s)	47.45 ± 19.79	31.76 ± 10.64		−2.353	0.018
PI (dB)	9.10 ± 11.25	18.48 ± 33.30		3.111	0.001
AUC (dB s)	249.33 ± 565.54	1126.95 ± 2076.72		4.544	<0.001
MTT (s)	28.54 ± 17.01	33.86 ± 7.62	−1.275		0.213
T₁/₂ (s)	22.00 ± 14.93	47.29 ± 13.68	0.0985		<0.001
Tr (s)	2.69 ± 11.12	12.93 ± 4.78		3.030	0.002
**SWE** (Young's modulus value)					
Cortex					
Mean (kPa)	66.36 ± 20.06	18.17 ± 3.98	10.593		<0.001
Min (kPa)	54.19 ± 16.79	11.29 ± 4.48	11.038		<0.001
Max (kPa)	78.00 ± 25.24	24.16 ± 3.77	9.436		<0.001
SD (kPa)	6.36 ± 2.88	3.28 ± 0.88	4.567		<0.001
Medulla					
Mean (kPa)	57.63 ± 17.98	19.22 ± 2.94	9.432		<0.001
Min (kPa)	47.45 ± 15.08	14.77 ± 3.79	9.400		<0.001
Max (kPa)	67.40 ± 21.79	23.26 ± 2.88	8.980		<0.001
SD (kPa)	5.11 ± 1.88	2.20 ± 1.01	6.084		<0.001
sinus					
Mean (kPa)	40.39 ± 32.47	17.21 ± 3.38		−4.734	<0.001
Min (kPa)	32.32 ± 23.49	14.47 ± 3.51		−4.815	<0.001
Max (kPa)	48.69 ± 39.21	19.43 ± 3.42		−4.896	<0.001
SD (kPa)	4.09 ± 3.61	1.20 ± 0.46		−4.518	<0.001
MRV of thrombosis					
Mean (kPa)	9.52 ± 3.49	–			
Min (kPa)	4.24 ± 4.56	–			
Max (kPa)	16.85 ± 6.71	–			
SD (kPa)	2.73 ± 2.41	–			

MRV: main renal vein; MRA: main renal artery; CSA: Curve slope of ascending; TTP: Time to peak; PI: Peak intensity; AUC: Area under the curve; MTT: Mean transit time; T_1_/_2_:Time from peak to half value; Tr: Rise time.

Logistic regression analysis identified four variables associated with the presence of AKI in the clinical model: TP, ALB, BUN, and CRE. The strength of association ranked: CRE > TP > ALB > BUN, with BUN yielding the highest area under the curve (AUC) of 0.780. For the CUS model, three variables were selected: volume, cortical thickness, and width of the MRV, with the association strength, ranked as volume > cortical thickness > width of the MRV. The width of the MRV exhibited the highest AUC of 0.944. In the CDFI + PW models, eight variables were selected: MSA, MSV, ISA, ISV, ILA, ILV, AA, and AV with the following association strengths: AA > ISA > MSV > MSA > AV > ISV > ILA > ILV. The highest AUC in this model was observed for ISV (AUC = 0.625). For CEUS, six variables were selected: cortex TTP, cortex T_1_/_2_, medulla T_1_/_2_, sinus MTT, sinus T_1_/_2_, sinus Tr. The association strength was ranked as: sinus T_1_/_2_ > medulla T_1_/_2_ > sinus Tr > cortex T_1_/_2_ > cortex TTP = sinus MTT. The highest AUC in this model was observed for cortical TTP (AUC = 0.683). In the SWE model, cortex min and medulla mean were selected, with association strength as follows: cortex min > medulla mean. The highest AUC in this model was observed for the medulla (AUC = 1.000). Further details are provided in Table 3.

**Table 3.** Logistic regression and ROC curve analysis.

	Logistic regression			AUC
	Selected variables	*p*	OR	Area
Clinical	TP	0.477	0.924	0.467
	ALB	0.524	0.895	0.413
	BUN	0.158	0.222	0.780
	CRE	0.394	1.022	0.600
CUS	volume	0.303	0.879	0.868
	cortical thickness	0.602	0.580	0.706
	width of MRV	0.120	0.362	0.944
CDFI and PW (Vmax)	MSA	0.999	1.443	0.000
	MSV	1.000	1.485	0.000
	ISA	1.000	1.613	0.000
	ISV	1.000	1.035	0.625
	ILA	1.000	0.894	0.000
	ILV	1.000	0.397	0.010
	AA	1.000	9.413	0.560
	AV	0.999	1.436	0.000
CEUS	cortex TTP	0.983	0.000	0.683
	cortex T₁/₂	0.981	0.001	0.218
	medulla T₁/₂	0.979	62625.293	0.120
	sinus MTT	0.979	0.000	0.405
	sinus T₁/₂	0.979	7356623798	0.503
	sinus Tr	0.981	24985.855	0.222
SWE	cortex min	0.977	789.36	0.982
	medulla mean	0.974	0.000	1.000

MSA: main stem artery; MSV: main stem vein; ISA: intersegment artery; ISV: intersegment vein; ILA: interlobar artery; ILV: interlobar vein; AA: arcuate artery; AV: arcuate vein.

### 3.3. Radiomic feature extraction, selection, and model construction

**Figure 2.**
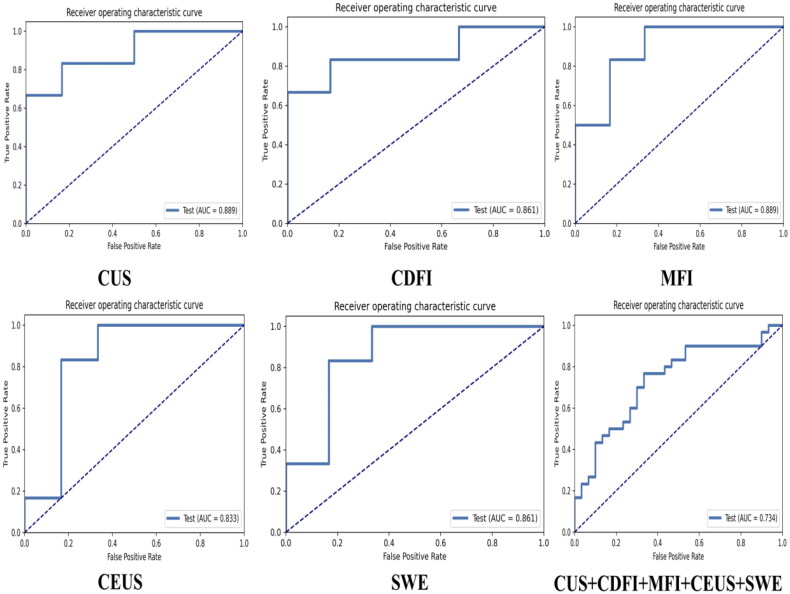
ROC Curves for CUS, CDFI, MFI, CEUS, SWE and CUS+CDFI+MFI+CEUS+SWE radiomic model in the testing cohort.

For the CUS, CDFI, MFI, CEUS, SWE, and CUS+CDFI+MFI+CEUS+SWE models, a total of 944 radiomic features were extracted from ultrasound images, and the models were based on 13 (CUS), 5 (CDFI), 4 (MFI), 1 (CEUS), 1 (SWE), 14 (CUS+CDFI+MFI+CEUS+SWE) features, respectively. These yielded the following AUCs and accuracies: 0.889 and 0.833 (CUS), 0.861 and 0.833 (CDFI), 0.889 and 0.833 (MFI), 0.833 and 0.833 (CEUS), 0.861 and 0.833 (SWE), 0.734 and 0.717 (CUS+CDFI+MFI+CEUS+SWE). Optimal model names were as follows: Mean-PCA-RFE-13-AE, MinMax-PCC-Relief-5-AE, MinMax-PCC-Relief-4-RF, Zscore-PCC-RFE-1-AE, Mean-PCA-Relief-1-LDA, and Zscore-PCA-Relief-14-LRLasso. Refer to Table 4 and Figure 2 for performance and ROC curve visualization.

**Table 4.** Performance of radiomic model in the testing cohort.

	CUS	CDFI	MFI	CEUS	SWE	CUS+CDFI+MFI+CEUS+SWE
AUC	0.899	0.861	0.899	0.833	0.861	0.734
ACC (%)	83.33	83.33	83.33	83.33	83.33	71.67
NPV (%)	83.33	83.33	83.33	83.33	83.33	74.07
PPV (%)	83.33	83.33	83.33	83.33	83.33	69.70
SEN (%)	83.33	83.33	83.33	83.33	83.33	76.67
SPE (%)	83.33	83.33	83.33	83.33	83.33	66.67
Model name	Mean-PCA-RFE-13-AE	MinMax-PCC-Relief-5-AE	MinMax-PCC-Relief-4-RF	Zscore-PCC-RFE-1-AE	Mean-PCA-Relief-1-LDA	Zscore-PCA-Relief-14-LRLasso

ACC: accuracy; AUC: area under the curve; NPV: negative predictive value; PPV: positive predictive value; SEN: sensitivity; SPE: specificity.

The composition of Model name: Normalizer-Dimension Reduction-Feature Selector-Feature Number-Classifier.

### 3.4. Pathological results

All 20 experimental kidneys exhibited varying degrees of renal histopathological injury. Based on KHIS, 2 cases were classified as mild, 15 as moderate, and 3 as severe. Thrombosis was observed in the main renal vein in 5 cases, and in both the main renal vein and corticomedullary junction in 15 cases.

## 4. Discussion

Acute renal vein thrombosis (ARVT) is a rare yet severe condition that leads to acute kidney injury (AKI), characterized by sudden renal dysfunction, following the obstruction of renal venous return. Its incidence has been increasing annually in recent years [17]. Pathophysiologically, ARVT involves interactions between altered hemodynamics, thrombus formation, and inflammation, manifesting in clinical symptoms such as flank pain, hematuria, and proteinuria, all of which may indicate renal venous obstruction and subsequent ischemia [18,19]. Rapid renal deterioration often ensues, potentially progressing to acute renal failure. In patients with nephrotic syndrome, complications like steroid-resistant proteinuria and pulmonary embolism may co-occur, which can further complicate their clinical status [20]. Given that ARVT can lead to life-threatening complications, such as acute renal failure and pulmonary embolism, early detection, and intervention are essential to prevent irreversible renal damage; however, due to its rarity, especially in cases of idiopathic renal venous thrombosis, it is often underdiagnosed or misdiagnosed [4]. In this study, animal models, particularly in rabbits, are valuable for advancing our understanding of pathophysiology and developing new therapeutic approaches. Rabbit models are particularly useful as the left renal vein is longer and more accessible than the right renal vein, enabling detailed experimental observations of thrombosis-related renal injury and its progression to acute kidney injury. In this study, complete renal vein occlusion was induced *via* ligation, achieving a mean stenosis rate of 95.6 ± 3.9%. This model successfully reproduced thrombotic injury patterns and served as a foundation for exploring the mechanisms underlying renal venous thrombosis and its associated renal injury.

In this study, we utilized various advanced ultrasonographic imaging techniques to investigate the effects of ARVT on renal hemodynamics and morphology. Prior studies [19] indicate that renal injury becomes evident as early as 30 min post-occlusion, with an increase in renal tissue injury scores. Additionally, Reyers’ research [21] indicated that the thrombosis rate in deep veins after surgical ligation is between 60% and 80% within 2 h. In this study, we found that within 30 min of surgical ligation of the left renal vein, macroscopic structural changes occurred in the left kidney, including enlargement and generalized congestion. Two hours later, thrombus formation was observed in the main trunk of the left renal vein using two-dimensional ultrasound (100% success rate). Therefore, we recommend collecting various ultrasound imaging data after 2 h for optimal results. The results from CUS, CDFI, MFI, CEUS, and SWE demonstrated significant changes in both the structure and function of the kidneys in the experimental group compared to the control group. The CUS and CDFI identified venous dilation and absence of flow signals, suggesting severe venous obstruction, with the thrombi appearing hypoechoic within the renal venous lumen. SWE characterized these thrombi as soft, fresh, clots with a low Young’s modulus (9.52 ± 3.49 kPa). Moreover, the absence of blood flow in the main renal vein further confirmed venous return obstruction and impaired renal perfusion. This finding aligns with that of a previous study [22], showing that venous thrombosis causes blood stagnation and acute kidney injury is characterized by enlarged kidneys, increased cortical thickening, and echogenicity. Logistic regression analysis also revealed significant changes in renal volume, cortical thickness, and renal venous diameter following ARVT-induced AKI. Furthermore, PW Doppler further indicated a significant decrease in peak systolic velocity of the renal arteries and veins in the experimental group compared to the control group (*p* < 0.001). This reduction in flow velocity, associated with a high-resistance vascular pattern and decreased perfusion, strongly indicates severe renal blood flow impairment following acute renal venous thrombosis. CDFI, MFI, and, CEUS each demonstrated specific advantages in assessing renal blood flow. CDFI was particularly effective in evaluating blood flow in the major renal vasculature, including the renal arteries, veins, and their branches. MFI, a novel ultrasound-based technique, offers the unique capability of detecting microvascular blood flow in the kidneys, displaying intricate microcirculatory dynamics [23,24]. This makes MFI valuable for detecting early changes in renal microcirculation before a significant functional decline occurs, thereby offering opportunities for early intervention by injecting microbubble contrast agents into the body and imaging the resulting echoes, which enhances the display of renal blood flow and perfusion [25]. Given the rich vascular structure of normal renal parenchyma, contrast agent microbubbles pass through the renal microcirculation without entering the renal tubules or interstitium, making CEUS a safe and effective method for assessing renal perfusion [26]. This technique is particularly advantageous in visualizing blood flow in the cortical microvessels and the finer microcirculatory structures. Through the enhancement effect of the contrast agent, CEUS provides a comprehensive evaluation of renal perfusion from large vessels to microvessels, enabling the detection of perfusion defects, which is crucial for the diagnosis of AKI, chronic kidney disease, and renal ischemia. By observing the dynamic distribution and clearance of the contrast agent, CEUS can reflect both renal blood flow and tissue function [27,28]. In this study, the CDFI assessment indicated that 20 cases in the experimental group were rated as grade III, MFI demonstrated 6 cases as grade II, and 14 cases as grade I, while CEUS showed 18 cases rated as grade II and 2 cases rated as grade III. These findings further support the hypothesis that ARVT induces a state of reduced renal microcirculatory perfusion, which is a key factor in the development of AKI. Additionally, CEUS can quantify changes in renal microcirculation after acute renal venous thrombosis using time-intensity curves. The parameters evaluated in this study included CSA, TTP, PI, AUC, MTT, T_1_/_2_, and Tr, where TTP and MTT reflect the clearance rate of microbubbles by the kidneys, PI indicates local tissue blood volume; AUC is related to renal blood volume, and CSA reflects the rate at which microbubbles enter the renal vasculature. Compared to the control group, the experimental group exhibited significant reductions in blood flow to the cortex, medulla, and sinus, with no statistically significant differences observed in CSA and Tr of the cortex, as well as TTP of the medulla and MTT of the sinus (*p* > 0.05), while other parameters showed significant differences (*p* < 0.05). Logistic regression analysis indicated that TTP of the renal cortex, T_1_/_2_ of the renal cortex, T_1_/_2_ of the renal medulla, MTT of the renal sinus, T_1_/_2_ of the renal sinus, and Tr of the renal sinus were of significant diagnostic value for AKI following ARVT. These findings indicate that ultrasound imaging techniques are crucial for the early detection and assessment of renal microcirculatory changes related to ARVT. They also show promise for clinical use in the diagnosis and management of AKI.

In the model of acute renal vein obstruction, SWE, an advanced imaging technique, is capable of providing real-time, quantitative assessment of changes in renal tissue stiffness. In this study, SWE was used to evaluate different regions of the kidney following acute renal vein obstruction. The results showed that Young’s modulus values for the cortex, medulla, and sinus in the experimental group were significantly higher than those in the control group (*p* < 0.001). These findings indicate that acute renal vein obstruction typically leads to hemodynamic abnormalities in the kidney, including impaired renal venous return, localized edema, and elevated intra-renal pressure, among other physiological and pathological changes [29]. These factors can alter the structural and mechanical properties of the renal tissue, subsequently affecting its elastic characteristics. Specifically, in the cortex, medulla, and sinus regions, acute venous congestion may increase stiffness through several mechanisms [29]: First, the renal cortex, as the primary functional region of the kidney is highly influenced by blood supply. Obstruction of venous return could lead to localized edema and changes in the extracellular matrix thereby increasing stiffness in this region. Second, the renal medulla has relatively poor blood supply, and during acute venous congestion, this region may experience more significant hemodynamic changes resulting in alterations to its mechanical properties. Lastly, the renal sinus, due to its structural characteristics (e.g., a large amount of adipose tissue and blood vessels), is more sensitive to changes in venous pressure, potentially showing increased stiffness as well. Notably, in this study, the renal cortex exhibited the highest Young’s modulus values and showed the most significant changes, which may reflect more severe early damage to the renal cortex during acute renal vein obstruction, or a higher sensitivity of this region to venous congestion. This phenomenon is likely related to the critical role of the renal cortex in kidney function and blood perfusion. Obstruction of venous return could lead to cellular edema, increased interstitial pressure, and local microvascular circulation impairment in the cortex thereby resulting in increased stiffness. In contrast, the changes in Young’s modulus in the renal medulla and renal sinus were relatively smaller, which may be attributed to the differing hemodynamic characteristics and structural properties of these regions. For instance, the renal medulla, with its relatively sparse vascular supply, may respond more slowly to reduced blood flow, and its internal structure may better buffer pressure changes. Meanwhile, the renal sinus, due to the abundant fat and the relatively lower proportion of functional renal tubule tissue, may exhibit a more gradual response to changes in blood flow. As a result, in the experimental group, the elasticity of the renal cortex was markedly higher than that of the renal medulla, with the renal sinus showing the lowest elasticity, consistent with prior research.

Radiomics is a technique that extracts a large number of quantitative features from medical images for analysis and has demonstrated significant advantages in early diagnosis and prognosis prediction in recent years, which include noninvasive and reproducible multidimensional information extraction, the provision of objective quantitative data, early detection, and ongoing disease monitoring [30–33]. In this study, we explored how radiomic features from different ultrasound imaging techniques, specifically CUS, CDFI, MFI, CEUS, and SWE, can predict AKI caused by ARVT. Through the extraction of 944 radiomic features from ultrasound images and the application of various machine-learning models for analysis, we identified the significant potential of ultrasound radiomics in the early detection of AKI. The CUS, CDFI, MFI, CEUS, SWE, and CUS+CDFI+MFI+CEUS+SWE models were constructed using 13 (CUS), 5 (CDFI), 4 (MFI), 1 (CEUS), 1 (SWE), 14 (CUS+CDFI+MFI+CEUS+SWE) features, respectively. The corresponding optimal AUC values obtained on the test dataset were 0.889, 0.861, 0.889, 0.833, 0.861, and 0.734 for each model, respectively. These results underscore the high diagnostic value of radiomic features in predicting AKI caused by ARVT. Notably, models such as Mean-PCA-RFE-13-AE, MinMax-PCC-Relief-5-AE, MinMax-PCC-Relief-4-RF, and Mean-PCA-Relief-1-LDA demonstrated high sensitivity and specificity in differentiating between AKI and non-AKI cases. Although the AUC values for the Z-score-PCC-RFE-1-AE and Zscore-PCA-Relief-14-LRLasso models showed some decline, their accuracy remained consistent at approximately 0.833 on the test set, indicating the stability and reliability of these models for practical clinical applications. Even in cases with significant variability in ultrasound image quality, the models maintained strong performance. From the perspective of feature selection, the application of RFE and Relief played a crucial role in enhancing model performance by reducing redundant features and simplifying model complexity while maintaining high predictive accuracy. Particularly, the dimensionality reduction through PCA contributed to improving model generalization and helped mitigate overfitting. Furthermore, Z-score normalization enhanced the adaptability of the models to diverse datasets, thereby improving stability. The integration of multimodal imaging features represents another strength of this study. By combining texture, shape, and intensity, the models provided a more comprehensive reflection of the pathological changes associated with ARVT-induced AKI. Although the combined models exhibited lower AUC and accuracy than the single models, this may be attributed to the early exploratory nature of the study and the relatively small sample size, which will require further expansion for additional exploration. Recently, deep learning has found increasing applications in image-based medical diagnostics. It possesses the ability to directly extract the most salient features from original image pixels, boasts a more intricate and easily scalable architecture, eliminates the need for manual definition of intermediate steps, and can develop models that more accurately depict lesions than those created by radiologists. Multimodal studies leveraging deep learning can harness the abundant information embedded in multimodal data, leading to enhanced outcomes [34,35]. Therefore, future research should delve deeper into the relationship between multimodal ultrasound techniques that utilize deep learning and single-mode deep learning approaches for this disease.

Despite the significance of this study, there are some limitations must be acknowledged. (1) Due to the rarity of AKI secondary to ARVT, the sample size of the animal model in this study was relatively small, which may have affected the generalizability of the results. Further studies with larger sample sizes are required for more robust analyses; (2) The animal model used may not fully replicate the complex pathological changes in AKI following ARVT in humans, further validation of its accuracy and applicability in real-time clinical settings is required; (3) This study is still in its preliminary stage, and the radiomic data were derived from a small-scale animal study, which may limit the external validation and clinical applicability of the model. There are plans to increase the sample size and incorporate different animal models in future research to validate our findings and enhance the validity of the acute kidney injury model; (4) The left and right kidneys of the same New Zealand white rabbit were used as the experimental and control groups, respectively. This approach may have some impact on the final results; however, it helps reduce the influence of individual differences, making the comparison more direct. However, further analysis with a larger sample size is needed to minimize the impact of such variations.

## 5. Conclusion

In conclusion, the rabbit model of renal venous thrombosis successfully reproduced the pathophysiological characteristics of acute renal venous thrombosis and its associated kidney injury and provides a crucial platform for advancing our understanding of the pathophysiology of ARVT. The model highlights the significant impact of ARVT on renal hemodynamics and morphology, as revealed by various advanced ultrasound imaging techniques. These findings underscore the potential clinical application of ultrasound in the early diagnosis of AKI due to ARVT. While traditional clinical models showed limited diagnostic value in the early stages, radiomic models derived from ultrasound data, combined with machine learning techniques, demonstrated superior diagnostic performance. Radiomics has proven to be an effective tool for predicting acute AKI following ARVT, demonstrating strong potential for early detection. Future research utilizing independent datasets is needed to further validate the robustness and generalizability of radiomic-based predictive models in clinical practice and should include a comparative analysis with standard diagnostic methods, such as renal venography, contrast-enhanced CT, and MRI, to explore the correlation and diagnostic value of radiomics combined with multimodal ultrasound techniques against these standard methods.
